# Ten-Year Poverty Alleviation Effect of the Medical Insurance System on Families With Members Who Have a Non-communicable Disease: Evidence From Heilongjiang Province in China

**DOI:** 10.3389/fpubh.2021.705488

**Published:** 2021-09-09

**Authors:** Qi Xia, Lichun Wu, Wanxin Tian, Wenqing Miao, Xiyu Zhang, Jing Xu, Yuze Li, Baoguo Shi, Nianshi Wang, Huiying Yang, Zhipeng Huang, Huiqi Yang, Ye Li, Linghan Shan, Qunhong Wu

**Affiliations:** ^1^Harbin Medical University, Harbin, China; ^2^Department of Stomatology, Heilongjiang Provincial Hospital, Harbin, China; ^3^Department of Urology, Heilongjiang Provincial Hospital, Harbin, China; ^4^Department of Medicine, Jiamusi University, Jiamusi, China; ^5^Department of Economics, School of Economics, Minzu University of China, Beijing, China; ^6^The Department of Hospital Offices, Nanjing Medical University, Wuxi, China; ^7^The Second Affiliated Hospital of Harbin Medical University, Harbin, China

**Keywords:** catastrophic health expenditure, healthy poverty, medical insurance, poverty alleviation, non-communicable diseases

## Abstract

**Aims:** Non-communicable diseases (NCD) drag the NCD patients' families to the abyss of poverty. Medical insurance due to weak control over medical expenses and low benefits levels, may have actually contributed to a higher burden of out-of-pocket payments. By making a multi-dimensional calculation on catastrophic health expenditure (CHE) in Heilongjiang Province over 10 years, it is significant to find the weak links in the implementation of medical insurance to achieve poverty alleviation.

**Methods:** A logistic regression was undertaken to predict the determinants of catastrophic health expenditure.

**Results:** The average CHE of households dropped from 18.9% in 2003 to 14.9% in 2013. 33.2% of the households with three or more NCD members suffered CHE in 2013, which was 7.2 times higher than the households without it (4.6%). The uninsured households with cardiovascular disease had CHE of 12.0%, which were nearly 10% points lower than insured households (20.4–22.4%). For Medical Insurance for Urban Employees Scheme enrolled households, the increasing number of NCD members raised the risk of impoverishment from 3.4 to 20.0% in 2003, and from 0.3 to 3.1% in 2008. Households with hospital in-patient members were at higher risk of CHE (OR: 3.10–3.56).

**Conclusions:** Healthcare needs and utilization are one of the most significant determinants of CHE. Households with NCD and in-patient members are most vulnerable groups of falling into a poverty trap. The targeting of the NCD groups, the poorest groups, uninsured groups need to be primary considerations in prioritizing services that are contained in medical insurance and poverty alleviation.

## Introduction

Non-communicable diseases (NCD) have become a leading cause of death globally. In 2012, 38 million people died of chronic diseases worldwide, accounting for 68% of all deaths (1). Almost three quarters of all NCD deaths (28 million) occurred in low- and middle-income countries. In China, the total NCD mortality rate of national inhabitants was 533/10 million (9.85 million people) in 2012, and accounted for 86.6% of all deaths in China. The main causes of death in 2012 were cardiovascular disease, cancer and chronic respiratory, and they jointly accounted for 79.4% of the total number of deaths (2). The prevalence of NCD among Chinese citizens has risen from 123.3 per thousand in 2003 to 245.2 per thousand in 2013 (3, 4); the incidence rate has doubled in 10 years with a concomitant negative impact on productivity and economic development.

The more than 300 million NCD patients are rapidly becoming an increasing burden on social wealth and medical resources. The medical expenses for NCD account for 70% of total medical expenditure in China (5). Premature death and loss in productivity caused by chronic disease not only translate into economic losses for society, but the serious economic burden placed on families may even drag the NCD patients' families to the abyss of poverty. International evidence has shown that the rising burden of NCDs has impeded poverty reduction initiatives in low- and-middle income countries.

As a chronic condition, NCDs can lead to devastating, long-term economic consequences for individuals and their households, that not only impact adherence to the long-term therapy but also impair the NCD's quality of life. Households with NCD patients are thus trapped in the dilemma of choice: opting for long-term treatment will push their households into poverty, abandoning treatment will have an adverse effect on the health and economic prospects for both the individual and their households (6). Addressing the economic burden that NCD place on households is an important step in efforts to alleviate global poverty and achieve the UN's Sustainable Development Goals (7).

Medical health insurance is seen as an effective approach to reduce the economic burden of disease. China has attained a universal national health system with 96% of national residents covered by three basic health insurance schemes in 2011. China's basic medical insurance system includes the Medical Insurance for Urban Employees (MIUE) scheme designed exclusively for urban employees, the Medical Insurance for Urban Residents scheme (MIUR) designed for urban residents who are not covered by the MIUE, and the New Cooperative Medical Scheme (NCMS) that provides cover for rural citizens. However, due to weak control over medical expenses and low benefit levels, the increased breadth of coverage may have actually contributed to a higher burden of out-of-pocket payments.

The MIUE was first launched in Heilongjiang in 1999, marking the establishment of basic medical insurance system (8). From 2003 to 2013, the number of people participating in MIUE increased from 4.352 to 5.565 million, an increase of 30.2% in 10 years (3, 4). In addition, Heilongjiang began to carry out the pilot of the NCMS scheme in the second half of 2003. By the end of 2012, the participation rate of the NCMS in Heilongjiang Province reached 99.3%. From 2003 to 2013, the number of population participating in basic medical insurance system in Heilongjiang has been greatly increased, basically achieving universal coverage of the population width (8).

Previous studies have indicated that households with members who have chronic diseases suffer higher financial risks than other households. Health insurance scheme benefits have an unintended inequitable distributive effect in terms of catastrophic health expenditure (CHE) incidences. A high incidence rate of health expenditure on emergency events reveals the inadequacies of medical health insurance schemes in achieving their goal of protecting household finances. Therefore, our study makes a multi-dimensional calculation on emergency health expenditure and impoverishment caused by medical expenses in Heilongjiang Province over 10 years, in order to find the weak link in the implementation of the medical insurance system.

## Methods

### Data Source and Sampling Method

Data were obtained from the Third, Fourth, and Fifth Health Service Surveys of Heilongjiang Province, which fall under the National Health Service Survey (NHSS, 2003, 2008, and 2013). Heilongjiang Province is located in northeast China, and has a population of 38.1 million and covers an area of over 473,000 km^2^. At the end of 2013, the per capital GDP was 37,697 Yuan, ranking it among the poorest provinces in China. As one of the most authoritative health surveys in China, the NHSS is conducted every 5 years by the Ministry of Health of China. A multistage stratified cluster random sampling method was used to represent the whole population. The subjects of this survey are randomly re-sampled every year as follows: using 10 socio-economic indicators, municipalities of Heilongjiang Province were ranked into five groups, and 30 urban streets and 30 rural townships were randomly selected. In each randomly sampled township, two administrative villages/communities were randomly selected. Finally, 30 households from each village/community were invited to participate in the survey in 2003, and 60 households were invited in 2008 and in 2013.

### Data Collection and Quality Control

To guarantee the quality of the sampled data, a face-to-face interview was conducted by qualified investigators for every member of the selected household. The survey questionnaire, developed by the Center for Health Statistics and Information of the Ministry of Health of China, covered the contents as follows: the socio-economic and demographic characteristics of the households, including annual income, out-of-pocket health expenditure, household food consumption expenditure, and the age, sex, education, employment, and insurance status of the head of the household; the health status of the household included information such as household members with illnesses during the previous 2 weeks, household members suffering from cardiovascular disease, diabetes, chronic respiratory disease, and cancer over the past 6 months; and the health service utilization of the households. During the whole process of data collection, quality control was implemented by specialized survey supervisors and 5% of the sampled households were revisited to check the accuracy of the data, the consistency rates of which were over 95%. The Myer's Index, the DELTA dissimilarity coefficient, and the GINI concentration ratio showed good consistency in age and household size between the survey sample and the general population in 2003, 2008, and 2013 (9).

After the process of data cleaning, a small number of sampled households with incomplete or anomalous data were excluded. A sample of 3,820 households with 11,501 individuals in 2003, 5,411 households with 15,571 individuals in 2008, and 5,289 households with 14,431 individuals in 2013, were finally included in the analysis.

### Ethics Statement

All the participants obtained and signed an informed consent document prior to data collection. The Ethics Review Board of Harbin Medical University has approved this study.

## Statistical Analysis

### Calculation of Catastrophic Health Expenditure

The study adopted the method developed by the WHO to calculate catastrophic health expenditure. The following key variables were included in the data process: out-of-pocket health expenditure payments (OOP) paid by household for medical expenses without any compensation; the poverty line, which was measured as food expenditure's share of total household consumption expenditure that fell between the 45th and 55th percentiles of the entire sample; household subsistence expenditure (SE), which was calculated as the poverty line multiplied by the standard household size (actual household size)^0.56^; and the household's capacity to pay (CTP), which was defined as the non-subsistence expenditure of a household (the total household consumption expenditure (EXP) minus subsistence spending). However, some households may report food expenditure (FOOD) that is lower than SE. This indicates that the household's FOOD is less than the estimated poverty standard for that country. Such a situation could also be due to the fact that the reported FOOD in the survey does not consider food subsidies, coupons, self-production and other non-cash means of food consumption. In this particular case the non-food expenditure is used as non-subsistence spending. However, whenever food expenditure was less than subsistence expenditure (SE >FOOD), the capacity to pay was identified as total expenditure minus food expenditure (CTP = EXP–FOOD); On the contrary (SE < = FOOD), the capacity to pay was identified as total expenditure minus subsistence expenditure (CTP = EXP–SE). CHE was finally defined as OOP payment for health care ≥40% of household CTP (10).

### Determinant Analysis on Catastrophic Health Expenditure

A logistic regression was undertaken to predict the determinants of catastrophic health expenditure. The independent variables entered into the model included:

1) The demographic characteristics of household: age, sex, education level of the head of the household; having members aged above 60 years old or below 5 years old; and household size;
2) The socio-economic status of the household: household economic level, which was ranked by equivalized per capita household consumption expenditure weighted with the standard household size; and the employment and insurance status of the head of the household;
3) The illness and health services condition: having household members with chronic disease, divided into cardiovascular disease, diabetes, chronic respiratory disease and cancer according to the recommendation of WHO; the number of household members with chronic diseases; having household members who are hospital in-patients, and whether any household member used medical in-patient services during the past year.

## Results

### Characteristics of the Respondents

Descriptive statistics were used for all the study variables analysis as showed in Table 1. The majority of the respondents were male (84.6, 84.0, and 86.3%) and had no tertiary education (90.1, 92.7, and 94.5%) at the time of the three NHSS in 2003, 2008, and 2013, respectively. From 2008 to 2013, the percentage of individuals with NCDs rose rapidly from 11.6 to 23.3%. This was especially evident among cardiovascular disease and diabetes patients where the number of patients suffering from cardiovascular disease and diabetes sharply increased by 3.7 and 3.3 times, respectively, during the 10 years. In 2013, 23.7% of the households had at least two or more members with chronic diseases, compared to only 11.8% in 2003. During the same period, the proportion of household heads without medical health insurance dropped sharply from 80.3 to 11.9%.

**Table 1:** Characteristics of respondents from the NHSS of Heilongjiang Province in 2003, 2008, and 2011.

**Year**	**2003**	**2008**	**2013**
	***N* (%)**	***N* (%)**	***N* (%)**
**Households**	3,820	5,411	5,289
**Individuals**	11,501	15,571	14,431
**Individuals with NCDs**	1,332 (11.6)	2,672 (17.2)	3,362 (23.3)
**Household residence**			
Urban	—	—	979 (18.5)
Rural	—	—	4,310 (81.5)
**Gender of household head**			
Male	3,230 (84.6)	4,545 (84.0)	4,566 (86.3)
Female	590 (15.4)	866 (16.0)	723 (13.7)
**Education level of household head**			
Illiterate	297 (7.8)	358 (6.6)	330 (6.2)
Primary school	925 (24.2)	1,420 (26.2)	1,785 (33.7)
Junior high school	1,781 (46.6)	2,551 (47.1)	2,420 (45.8)
Senior high school	439 (11.5)	690 (12.8)	467 (8.8)
Junior college or above	378 (9.9)	392 (7.3)	287 (5.5)
**Employment status of household head**			
Retired	450 (11.8)	759 (14.0)	394 (7.4)
Employed	2,863 (74.9)	3,479 (64.3)	4,274 (80.8)
Unemployed	507 (13.3)	1,173 (21.7)	621 (11.8)
**Health insurance status of household head**			
Medical insurance for urban employees scheme (MIUE)	753 (19.7)	1,286 (23.8)	564 (10.7)
Medical insurance for urban residents scheme (MIUR)	—	—	208 (3.9)
New cooperative medical scheme (NCMS)	—	2,693 (49.8)	3,886 (73.5)
Not insured	3,067 (80.3)	1,432 (26.4)	631 (11.9)
**Household annual income**	11623.35	16673.33	34029.26
**Household annual expenditure**	10403.18	13065.42	24529.02
**Household with member below 5 years old**	461 (12.1)	663 (12.3)	594 (11.2)
**Household with member above 60 years old**	869 (22.7)	1,496 (27.6)	1,746 (33.0)
**Household with in-patient member**	294 (7.7)	825 (15.2)	947 (17.9)
**Household size**			
>3	1,254 (32.8)	1,538 (28.4)	1,689 (31.9)
<=3	2,566 (67.2)	3,873 (71.6)	3,600 (68.1)
**Household including the type of chronic disease patients**			
Cardiovascular disease	365 (9.6)	861 (15.9)	1,866 (35.3)
Diabetes	424 (11.1)	982 (18.1)	1,949 (36.9)
Chronic respiratory disease	114 (3.0)	136 (2.5)	89 (1.7)
Cancer	0 (0)	31 (0.6)	20 (0.4)
**Households including chronic disease members**			
0	275 (72.0)	339 (62.8)	2,741 (51.8)
1	621 (16.3)	106 (19.7)	1,297 (24.5)
2	270 (7.1)	589 (10.9)	702 (13.3)
≥3	178 (4.7)	359 (6.6)	549 (10.4)

### Out-of-Pocket Payment and Catastrophic Health Expenditure

The average catastrophic health expenditure of households dropped from 18.9% in 2003 to 14.9% in 2013, while the impoverishment induced by medical expenses also dropped slightly from 4.1% in 2008 to 3.1% in 2013. Both trends for these two indicators had shown statistically significant differences in all three NHSS. The out-of-pocket payment had the biggest impact on households' capacity to pay with the rising number of NCD patients in all the three NHSS, especially in 2008 with the proportion of out-of-pocket payments rising from 20.8 to 47.3%. The incidence of catastrophic health expenditure was closely correlated with the NCD status of household. The more NCD members the households had, the higher the risk of occurring catastrophic health expenditure and impoverishment. In 2013, 33.2% of the households with three or more NCD members suffered incidences of catastrophic health expenditure, which was 7.2 times higher than the households without NCD members (4.6%) and 2.8 times higher than the households with 1 NCD member (11.7%). The impoverishment caused by self-financed medical expenses showed the same trend in all the three NHSS (see Table 2).

**Table 2:** Catastrophic health expenditure distribution in NCD households.

	**NCD status of household**				
	**HH without NCD member**	**HH with 1 NCD member**	**HH with 2 NCD members**	**HH with 3 or more NCD members**	**Total**
**2003**					
Average OOP (Yuan)	468.4	829.6	971.1	1161.6	920.6
Average CTP (Yuan)	3655.9	3980.6	7451.7	2455.1	4613.4
OOP/CTP (%)	12.8	20.8	13.0	47.3	20.0
HH with CHE (%)	7.4	17.9	27.8	37.1^**^	18.9
HH with IM (%)	3.2	4.8	4.4	12.9^**^	4.1
**2008**					
Average OOP (Yuan)	1373.8	2505.9	3354.6	4163.5	3049.3
Average CTP (Yuan)	9833.2	9873.0	10858.7	13280.5	10768.3
OOP/CTP (%)	13.9	25.4	30.9	31.4	28.3
HHs with CHE (%)	8.6	22.1	30.1	40.7^**^	20.9
HH with IM (%)	3.1	5.2	5.6	7.5^**^	4.0
**2013**					
Average OOP (Yuan)	1946.5	3367.1	5433.8	7362.8	4797.4
Average CTP (Yuan)	20067.9	19345.1	20256.7	20842.9	19918.9
OOP/CTP (%)	9.7	17.4	26.8	35.3	24.1
HHs with CHE (%)	4.6	11.7	23.6	33.2^**^	14.9
HH with IM (%)	1.3	3.9	5.3	7.6^**^	3.1

*OOP, out-of-pocket; CTP, capacity to pay; CHE, catastrophic health expenditure; IM, Impoverishment by medical expense; HH, households*.

** *P < 0.01 compared with the household with different NCD status*.

### Catastrophic Health Expenditure Distribution in Households With Different Types of NCD

We compared the incidence of catastrophic health expenditure among different NCD patients with different medical health insurance schemes (Table 3). Overall, in 2003 and 2008, the catastrophic health expenditure rates for households covered by medical health insurance were lower than those who were not covered. Especially for households with members suffering from cardiovascular disease, catastrophic health expenditure's proportion of total expenditure for those who were not covered by any insurance scheme was 31.4% in 2003 and 43.7% in 2008, respectively, which were, respectively, 3.6% points and 14.9% points higher than those covered by MIUE. The same trend happened in households with members with diabetes. A combination of several family-level risk factors, such as not having insurance and having NCD members, increased these households' vulnerability to catastrophic health expenditure. Households with diabetic members who did not belong to a medical health insurance scheme had catastrophic health expenditure rates of 48.0% in 2008, which was 1.78 times higher than households with members with diabetes but with MIUE.

**Table 3:** Distribution of catastrophic health expenditure for different types of NCD households in different health insurance schemes.

**Health insurance scheme of household head**	**Incidence of catastrophic health expenditure (%)**				
	**Cardiovascular disease**	**Diabetes**	**Chronic respiratory disease**	**Cancer**	**Total**
**2003**					
MIUE	27.8	32.1	27.3	—	28.7
Not insured	31.4^*^	31.8^**^	29.1	—	31.0
**2008**					
MIUE	28.8	26.9	52.9	56.3	30.9
NCMS	29.0	41.2	23.3	66.7	29.5
Not insured	43.7^**^	48.0^**^	34.5	16.7	43.0^**^
**2013**					
MIUE	20.4	25.0	33.3	—	21.6
MIUR	22.1	13.8	—	—	19.1
NCMS	22.4	26.4	40.0	52.6	24.1
Not insured	12.0^*^	15.6^*^	33.3	—	13.6^**^

*MIUE, Medical insurance scheme for urban employees; MIUR, Medical Insurance for urban residents; NCMS, New cooperative medical scheme for rural residents*.

* *P < 0.05*,

** *P < 0.01 compared with the household with different health insurance scheme*.

Since the development of a national medical health insurance system in 2013, the overall incidence of catastrophic health expenditure for all NCDs has declined. The rate of CHE for MIUE households declined from 30.9% in 2008 to 21.6% in 2013. However, it is still worth noting that uninsured households have a much lower incidence rate of catastrophic health expenditure compared to insured households. The uninsured households with members with cardiovascular disease had catastrophic health expenditure rates of 12.0%, which were nearly 10 percentage points lower than insured households (20.4–22.4%). Uninsured and suffering from NCD, should be used as a joint fragile factor to strengthen the vulnerabilities of the households to occurring catastrophic health expenditure. This demonstrated that uninsured households may choose to give up medical treatment and to avoid catastrophic health expenditure. Meanwhile, it cannot be ignored that households with medical health insurance and household members with cancer members had the highest catastrophic health expenditure rates (52.6–66.7%), which were much higher than the average rates (see Table 3).

### Distribution of Medical Impoverishment Distribution in Households With Different Numbers of NCD

Households with NCD members were associated with incidences of impoverishment induced by medical expenses. Households with a greater number of NCD members have a higher risk of incurring impoverishment due to medical expenses. For MIUE enrolled households, the increasing number of NCD members raised the risk of impoverishment from 3.4 to 20.0% in 2003 with a statistically significant difference (*P* < 0.01), and from 0.3 to 3.1 % in 2008. Since the development of the MIUE, the incidence of impoverishment caused by medical expenses showed a clear downward trend in NCD households, and by 2013 no household were trapped in impoverishment because of paying for medical expense due to the economic protection provided by the MIUE. Compared to the MIUE, the NCMS's cover was too insufficient to provide economic protection for the NCD households. In 2008, 5.3% of the NCMS households with 1 NCD member suffered from impoverishment, 7.2% for the households with two NCD members, and 9.8% for households with three or more NCD members (*P* < 0.01). After 5 years' development of the NCMS, incidences of impoverishment due to medical expenses have not significantly improved, and in 2013 still ranged between 5.0% for households with 1 NCD member to 10.7% for households with three or more members.

We next compared the same NCD members of households among different medical health insurance schemes. The results demonstrated that significant differences existed in the incidence of impoverishment among different medical health insurance schemes. Not being covered by medical health insurance was originally considered a high risk factor to impoverishment through unplanned medical expenses, however, for households with same number of NCD members, the impoverishment rates of the uninsured NCD households were not the highest compared to other insured NCD households. Especially in 2013, the NCMS enrolled NCD households had much higher impoverishment rates than the uninsured NCD households, the rates of which were 5.0%, 7.0%, and 10.7%, compared to 2.0%, 1.8%, and 1.7%, respectively, for uninsured households (see Table 4).

**Table 4:** Impoverishment by medical expense distribution in households with different numbers of NCD in different medical health insurance schemes.

	**Incidence of impoverishment by medical expense**			
	**HH without NCD member**	**HH with 1 NCD member**	**HH with 2 NCD members**	**HH with 3 or more NCD members**
**2003**				
MIUE	5.3	3.4	10.0	20.0^##^
Not insured	2.7^**^	5.4	2.9^*^	10.2^*^
**2008**				
MIUE	1.5	0.3	1.9	3.1
NCMS	3.9	5.3	7.2	9.8^##^
Not insured	2.9^**^	9.1^**^	6.3^*^	7.5
**2013**				
MIUE	0.0	0.0	0.0	0.0
MIUR	1.0	0.0	2.4	3.7
NCMS	1.5	5.0	7.0	10.7^##^
Not insured	1.1	2.0^**^	1.8^*^	1.7^**^

* *P < 0.05*,

** *P < 0.01 compared with the household with different health insurance scheme*.

# *P < 0.05*,

## *P < 0.01 compared with the household with different NCD status*.

### Determinants of Catastrophic Health Expenditure

The logistic regression model results showed that households headed by a retired or an unemployed person were more likely to experience catastrophic health expenditure compared to those headed by employed an employed person. Retired heads of households increased the odds of households suffering from CHE 2.05 times in 2003, 2.23 times in 2008 and 2.64 times in 2013. The economic level of the household was inversely associated with catastrophic health expenditure. The poorest households were more likely to incur catastrophic health expenditure than the wealthiest households, and this increased over time: 2.57 times more likely in 2003, 5.21 times in 2008, and 4.02 times in 2013. Households with hospital in-patient members were at higher risk of catastrophic health expenditure (OR: 3.10–3.56). Having more NCD members increased the odds of incurring catastrophic health expenditure in all the 3 years. Households with cancer patients were more likely to experience catastrophic health expenditure, 3.58 more in 2008, and 3.76 times more in 2013, than those households without cancer members. Having a larger family members appeared to be protective factor for a household to escape catastrophic health expenditure (see Table 5).

**Table 5:** Determinants of catastrophic health expenditure for NCD households.

**Variables**	**2003**	**95% CI**		**2008**	**95% CI**		**2013**	**95% CI**	
	**OR**	**Lower**	**Upper**	**OR**	**Lower**	**Upper**	**OR**	**Lower**	**Upper**
**Householder sex** (Male vs. Female)	1.39	0.91	2.11	1.26	0.93	1.71	1.29	0.93	1.81
**Householder education level**									
None vs. Junior college or above	1.74	0.86	3.50	3.06^**^	1.54	6.08	1.23	0.63	2.42
Primary vs. Junior college or above	1.19	0.64	2.24	2.98^**^	1.58	5.63	0.94	0.51	1.73
Junior high vs. Junior college or above	0.97	0.53	1.77	2.07^*^	1.11	3.84	0.77	0.43	1.40
Senior high vs. Junior college or above	1.28	0.63	2.59	1.62	0.81	3.25	0.66	0.34	1.27
**Householder employment status**									
Retired vs. Employed	2.05^**^	1.34	3.14	2.23^**^	1.47	3.38	2.64^**^	1.62	4.30
Unemployed vs. Employed	0.93	0.59	1.47	1.57^**^	1.12	2.19	2.64^**^	2.01	3.48
**Householder health insurance status** ^**a**^									
MIUR vs. MIUE	–	–	–	–	–	–	0.57	0.29	1.12
NCMS vs. MIUE	–	–	–	1.29	0.87	1.91	1.11	0.66	1.85
Not insured vs. MIUE	1.55^*^	1.02	2.36	1.24	0.87	1.77	0.68	0.37	1.25
**Family economic group quintile**									
1 vs. 5	2.57^**^	1.51	4.36	5.21^**^	3.29	8.26	4.02^**^	2.38	6.79
2 vs. 5	1.81^*^	1.06	3.10	2.63^**^	1.64	4.22	1.98^*^	1.15	3.41
3 vs. 5	1.45	0.87	2.42	2.12^**^	1.33	3.38	1.44	0.79	2.62
4 vs. 5	1.92^*^	1.13	3.24	1.48	0.92	2.40	1.07	0.55	2.07
**Family size** (≤3 vs. >3)	2.08^**^	1.44	3.02	2.11^**^	1.57	2.84	1.21	0.92	1.60
**Including member below 5 years old** (Yes vs. No)	0.96	0.50	1.84	1.39	0.88	2.21	0.63	0.35	1.15
**Including member above 60 years old** (Yes vs. No)	0.98	0.68	1.41	1.51^**^	1.17	1.95	1.07	0.83	1.39
**Including inpatient member** (Yes vs. No)	3.10^**^	2.09	4.61	3.56^**^	2.75	4.62	3.16^**^	2.50	3.99
**Family chronic disease amount**									
2 vs. 1	1.36	0.94	1.97	1.31^*^	1.01	1.71	1.84^**^	1.41	2.41
≥3 vs. 1	1.93^**^	1.26	2.95	1.73^**^	1.27	2.36	2.05^**^	1.53	2.76
**Including member suffering cardiovascular disease** (Yes vs. No)	1.41	0.99	1.98	1.24	0.98	1.57	1.22	0.93	1.61
**Including member suffering chronic respiratory disease** (Yes vs. No)	1.28	0.78	2.08	1.05	0.68	1.64	1.83^*^	1.08	3.11
**Including member suffering cancer** (Yes vs. No)	–	–	–	3.58^**^	1.54	8.28	3.76^**^	1.43	9.90
**Including member suffering diabetes** (Yes vs. No)	1.57	0.88	2.80	1.63^*^	1.08	2.47	1.30	0.96	1.77
**Constants**	0.02			0.01			0.02		

* *P < 0.05*;

** *P < 0.01*.

## Discussion

In our study, a systematic analysis was made of the incidence of catastrophic health expenditure and impoverishment by medical expense for NCD households in Heilongjiang Province by access survey data from 2003, 2008, and 2013. Although the catastrophic health expenditure rate showed a downward trend during the 3 years (18.9%, 20.9%, and 14.9%, respectively), the overall incidence rates were not only higher than the national average rates (11), but also higher than that in most developing counties (12, 13). The factors that drove catastrophic health expenditure NCD households into poverty over the 10 year period are summarized in subsequent sections:

### Economic Related-Demographic Factors

The results of the logistic regression showed that demographic factors such as education level, family size, employment status of the household head, having elderly family members, and the family's economic level all exert an influence on the risk of incurring catastrophic health expenditure. A higher education level, larger family size, and employment status are all protective factors, and consistent with the results described in other literatures (14, 15). Economic status could help to increase a household's financial capacity to resist CHE, however there are still a great number of households living under the poverty line. Heilongjiang Province, as a region had 2.11 million (8.4%) poor people in 2015, 2.7% higher than the national average (16). The problem of poverty caused by illness in the poor must to be urgently addressed and solved. Our study found that in 2003, the poorest group are 2.6 times more likely to be impacted by CHE than the richest group. This trend continued and become more severe in 2008, and only slightly relieved until 2013; however, the poor of Heilongjiang Province still face great challenges. Thus, devoting funds and government resources to appropriate poverty alleviation amongst the poor is key to alleviate inequality caused by CHE.

### High Healthcare Utilization

Healthcare needs and utilization are one of the most significant determinants of CHE. Our study revealed that households with chronic disease members and in-patient members are more at risk of falling into a poverty trap than others.

The impact of chronic diseases on a family's disease burden is mainly reflected in the two following aspects: First, households with more chronic disease members are more likely to incur economic difficulties. During the period under review (2008–2013), the incidence of catastrophic health expenditure for households with three or more NCD members was 3–7 times higher than that of households without NCD members, and twice as risky as the households with 1 NCD member. The incidences of catastrophic health expenditure for households with three or more NCD members were high ranging from 33.3% to 40.7%. Second, the different types of chronic disease also had an important impact on the occurrence of catastrophic health expenditure. Cancer, chronic respiratory disease, and diabetes contributed to the highest incidence of CHE and impoverishment in our study, which was in line with other studies. As the northernmost province in China, bad weather conditions, a diet high in salt and fat, as well as unhealthy lifestyle behaviors, all contributed to the high prevalence of NCD in Heilongjiang Province. Meanwhile, according to the data of the fifth health service survey of Heilongjiang Province, the prevalence of chronic diseases in Heilongjiang was 329.2% higher than the national average (245.2%), and significantly elevated compared with the forth survey in 2008 (3, 17). Participating in medical insurance will generate ex-ante moral hazard. Because people participate in medical insurance, the marginal price of medical services is lower than when they did not enroll in medical insurance. Therefore, there will be an ex-ante moral hazard, that is, people are more likely to ignore their own health and reduce their preventive investment in disease (18, 19). This is one of the factors contributing to the increase in the prevalence of chronic diseases in Heilongjiang. The rapidly increasing health care needs and medical resource utilization also aggravated pressure of health service consumption (20). The experience from high-income countries indicates that even in health systems that are well-recognized for having achieved universal health coverage, many households carry severe economic burdens, particularly those households with a family member who has long-term or chronic conditions and those in low socioeconomic groups. Even for the wealthy households with high economic levels and a strong capacity to pay, catastrophic health expenditures will occur due to the continuous nature of medical expenses for chronic disease and health services. The broad scope of formal patient co-payments and exemption or reduction of co-payments for vulnerable people are still not sufficiently effective to prevent catastrophic health expenditures in households with chronic disease (21).

### The Key Weakness of Medical Health Insurance Design for Effective Economic Protection

For vulnerable groups with overlapping risk factors, the medical insurance system does not give special preference. The targeting of the NCD groups, the poorest groups, and uninsured groups need to be primary considerations in prioritizing services that are contained in medical insurance schemes. However, our study finds that the medical health insurance schemes have not actually reduced the risk of catastrophic health expenditure and nor significantly relieved the financial burden of the registered chronic disease sufferers, which is similar to findings in other existing literatures (22–24). There are still 21.6% of the MIUE registered NCD households facing the dilemma of catastrophic health expenditure, and which is more severe for NCMS NCD households (24.1%). The performance of medical health insurance schemes in terms of financial protection is poorer for those households with chronic disease members. Especially for those households with cancer members, our results showed that in 2008 the incidence of CHE in different health insurance schemes were more than 50%, the highest incidences even more than 66.4%, and far higher than for any other NCD types. The weak performance of health insurance in financial protection may be caused by the high prevalence of chronic diseases among the elderly population and the corresponding medical expenditure pattern in policy design.

The medical insurance system has only lowered the threshold for Heilongjiang residents to use health services. The proportion of health benefits and reimbursement needs to be further expanded. Increasing the compensation level of financial protection and providing access to services of reasonable quality is an ever-present policy challenge, as exemplified in Thailand, where universal health coverage has been bolstered by subsequent extensions to benefit packages. Taking Harbin City, the capital city of Heilongjiang Province, as an example: the MIUE only covers 12 special chronic diseases with limited coverage for out-patient service and medicines. Further increasing the coverage of health benefits packages for NCD treatment and medicines is quite significant.

In addition, breaking inequity in economic protection between medical insurance schemes also needs to be resolved urgently in Heilongjiang Province. The incidence of impoverishment by medical expense for NCMS registered families in 2013 was high at 10.7%, much higher than the 3.7% for MIUR and the 0% for MIUE registered families. NCMS has the weakest economic protection ability, which is consistent with the research of other scholars (25–29). The NCMS covers a larger part of the rural population, who are also among the most vulnerable groups of NCD. Improving the economic protection of NCMS will help Heilongjiang to further achieve the poverty reduction effect of the medical insurance system.

## Data Availability Statement

The raw data supporting the conclusions of this article will be made available by the authors, without undue reservation.

## Author Contributions

QX, Ye Li, QW, and LS contributed to the conception of the manuscript and wrote the manuscript. WT, YuLi, NW, HuiyY, WM, and HuiqY collected the materials and data. QX, BS, XZ, and ZH contributed to the analysis or interpretation of data. LW and JX provided great help with providing suggestions and collecting the literature during the revision. All authors contributed to the article and approved the submitted version.

## Funding

This work was supported by the National Natural Science Foundation of China [grant nos. 72174047, 71874045, 71403073, 71333003, and 71804036], China Postdoctoral Science Foundation [grant no. 2016M590296], Heilongjiang Provincial Postdoctoral Science Foundation [grant no. LBH-Z14166], and Health and Family Planning Commission of Heilongjiang Province [grant no. 2014-427]. Humanities and Social Youth Foundation, Ministry of Education of the People's Republic of China [grant no. 19YJCGAT004].

## Conflict of Interest

The authors declare that the research was conducted in the absence of any commercial or financial relationships that could be construed as a potential conflict of interest.

## Publisher's Note

All claims expressed in this article are solely those of the authors and do not necessarily represent those of their affiliated organizations, or those of the publisher, the editors and the reviewers. Any product that may be evaluated in this article, or claim that may be made by its manufacturer, is not guaranteed or endorsed by the publisher.
